# A Memoir of the Means of Modifying the Forms of Living Bodies by Regimen

**Published:** 1844-08

**Authors:** 


Art. VII.—*A Memoir on the Means of Modifying the forms of Living Bodies by Regimen.* By M. Hippolyte Royer-Collard. Translated from the French of Dr. T. D. L. By John Barry, M.D., formerly Secretary of the Louisville Medical Society.

Anatomy and physiology have not made any considerable progress for the last twenty years except in the comparative study of man and the other organized species. Pathology has scarcely entered into this path. Hygiene, that branch of medicine which teaches the regulation of the life of man in such a manner as to obtain the free exercise of all his functions, and the complete development of all his faculties, hygiene is yet more backward in this respect than any of the other branches of the science; and yet more than any of them it may find in this study valuable and multiplied practical suggestions. Agriculture, the raising of live stock, the breaking and training of animals, has amassed during ages treasures of positive observations and the results of ready-made experiments. The most ignorant farmer in the country possesses knowledge which science neglects, an odd mixture of truth and errors, a rude product of an empiricism often gross, but sometimes inspired by genius. It is from this prolific source that M. Hippolyte Royer-Collard, Professor of Hygiene in the Parisian Faculty of Medicine, has drawn when proposing to modify artificially the forms of living bodies by means of regimen, a new and original problem in its application to the human species, and which became the subject of a memoir which the Academy of Medicine has highly extolled.

The author at first examines the vegetable kingdom and there finds undeniable proofs of the numerous manifestations of human power. Every body knows how a particular alimentation modifies the different parts of a plant. Here the stamens are changed into petals; then the same plant removed back into a poor uncultivated soil presents only simple flowers. There the operation of *pinching* turns from their destination the nutritive principles, so that fruit eyes are produced in place of leaves. In other cases roots are transformed into branches or branches into roots. The influence of manure on plants is well known. The root of the wild carrot is naturally slender and coriaceous. M. Vilmorin has discovered the manner of progressively developing and changing it so as to make it thick, juicy and fleshy.

Directing next his attention to the animal kingdom, M. Royer-Collard remarked how many important lessons are offered by the natural history of domestic animals, when these animals are examined in all the conditions in which they are daily placed by human industry. He cites the remarkable experiments of Bakewell, of Dishley, in England—the farmer who undertook to create, if the expression be allowable, a breed of oxen which should have no equals in the world. It is to him that is due the breed of the large horses now used for draught in London. It was he alone who obtained from his sheep, at Dishley, the union of the two qualities which theoretical agriculturists considered incompatable, viz: fineness of wool, with development of the fleshy parts. His art, at first purely empirical, became at length a system founded on principles. For the last fifty years the plan of Bakewell has been adopted throughout Europe. Regimen, considered as an art, has arrived at an astonishing degree of perfection. What animals are proper or improper for fattening are now known by certain signs. What conditions are necessary for bringing them to the required degree of *embonpoint*. What kind of food produces fat, or muscles; what aliment increases the milk of cows, and the wool of sheep. We can exactly measure for each animal the nourishment, the air, the light, the exercise, it requires, in order to be brought to such and such a state, or to be used for such and such employment.

After these numerous examples of the influence of alimentary regimen on plants and on animals, we ought assuredly to conclude that a similar influence might also be exercised on man. In order to give more value to this induction M. Royer-Collard looks for proof of it in the human species itself. In the earlier period of life the mode of alimentation of an infant determines the conformation of its osseous system. For a long time physicians have pointed out the danger of that mixed kind of alimentation in which pap or thickened milk is given to infants when there is a deficiency of the mother's milk. The greater part of the new-born children succumb under this regimen, and those which survive are exposed to rachitis.

In adult life or in the state of maturity, man may yet experience the influence of regimen on his organs. The training of boxers, of couriers, and of jockies presents remarkable examples. The information on this subject, which M. Royer-Collard has collected and which we are going to report, has been furnished him by the excellent work of Sinclair, and also from conversations with Lord Henry Seymore, the famous rearer of horses, and with Dr. Farral, an English physician of distinguished merit.

A boxer is a person of the age of 18 years at least, and of 40 at most. He enters the arena as far as the enclosure. His fists are closed but not armed. Placed before his adversary he waits for a given signal to commence the fight. Then the two champions let fly at each other vigorous fisty-cuffs from the head to the stomach. If one of the combatants be thrown down or stunned by the violence of the shock, nearly a minute of repose is allowed him; before the full minute elapses he arises and recommences the struggle, or if not, he is declared vanquished. Ordinary boxers during a combat of an hour and a half rest themselves in this manner from thirty to forty times. About fifteen years ago, in a fight between Maffey and McCarthy, which lasted four hours and forty-five minutes, one of the pugilists fell stunned 190 times. The duration of the combat is very variable; sometimes it does not exceed a few minutes, sometimes it extends to three, four, and even five hours. It will be easily conceived that serious injury and even death may be the result. Sad examples of this have been seen, but fortunately they are extremely rare. More frequently there remains after a few days no trace of blows so terrible—a remarkable circumstance. It may be said, without exaggeration, that in general the fights of boxers no more expose life or even health than a number of other pursuits which are not considered as being dangerous. Prodigious strength, singular address, an insensibility to blows which exceeds all belief, and at the same time, perfect health, these are the phenomena presented by these men, assuredly very different from those offered by the rest of their fellow men. How are they thus modified? It is by the preparations they have undergone, by the instructions they have received, the *training*, the *condition*, to speak their language; that is, by the regimen.

Previously to entering on 'condition' a boxer for example, weighs 128 pounds, at the end of a few days he weighs no more than 120. A short time after this he weighs again 128 pounds, sometimes more, sometimes less, according to his organization; but his muscles are extremely augmented in volume. His muscles are now firm, prominent, and very elastic to the touch; they contract with extraordinary force under the influence of the electric shock. The prominence of the abdomen disappears, the chest becomes expanded and obtrudes forward; respiration is full, deep and capable of long efforts. The skin is become very firm, glossy and transparent; much importance is attached to the latter condition. When the hand of a man fully prepared is placed before a lighted wax candle, the fingers appear of a beautiful rosy transparence. It is remarkable that the portions of the skin which cover the arm-pits and the sides of the chest do not tremble during the motions of the arms. They must on the contrary appear perfectly adherent to the subjacent muscles. This firmness of the skin resulting from the absorption of the fat opposes the extravasations serous or sanguineous which ordinarily are produced by blows. This is also an essential condition. In 1740 the famous boxer Broughton, after uninterrupted victories for sixteen years, lost the pugilistic crown for having one time omitted to submit to the necessary course of training. He received on the forehead a stroke which produced such a swelling that he was unable to open his eyes. He had become gross and plethoric; his skin had become soft and distended. Training would certainly have removed these inconveniences. The memorable fight which took place in 1814 between the boxer Cribb and the negro Molineaux is also adduced. Bets were made to the amount of £50,000 sterling. Molineaux being of colossal stature and herculean strength, refused to prepare himself. Cribb, on the contrary, found himself in unfavorable circumstances, he was fat and weighed 188 pounds. After three month's training under the direction of Captain Barclay, he was reduced to 152 pounds. The conflict was not long doubtful. The face of Molineaux soon became the seat of considerable tumefaction, and the struggle could not be continued.

The regimen of couriers during 'condition' is in some respects analogous to that of boxers. In other respects it is different, the object not being the same. In the latter case the prime object is to augment the strength; in the former it is wished to diminish the weight of the body, and at the same time to increase the power of respiration. For the courier the result of training is as certain as for the pugilist. A courier after two day's training diminished in weight 18 pounds, and after five days 25 pounds. A man who weighed 120 finds himself commonly reduced in 16 days, and even in a shorter time to 80 pounds. It is known with certainty what will be best from one day to another. At the close of such training, the courier is become not only less heavy but more strong and enduring. Previously to being prepared he could not run a mile without losing his breath, now he can easily run twenty-five miles. There are in England runners who make twenty-five miles backwards per day during six weeks. The runner Torensed has in the same manner gone from London to Brighton (62 miles) in eight hours. At another time he went 100 miles in twelve hours, running one half and walking the other.

With respect to jockies their regimen is far from being so favorable to health. It is proposed solely to lessen their weight, and this object can seldom be attained but at the expense of their strength. Therefore many of them sink early or late under the treatment, whilst boxers, whose lives are sober and regular, are remarkable for their longevity.

M. Royer-Collard concludes his memoir by making known the principles of training. The regimen, which is continued for a longer or a shorter period, according to the object in view, and the state of the person who is to be submitted to it, consists, in the case of boxers and couriers, of two distinct and successive operations. The trainers commence by depriving the body of the fat, and of the superfluous fluids contained in the cellular tissue. These objects are attained by purgatives, by sudorifics, and by low diet. They insist on the employment of these means much more in the case of the courier than in that of the boxer. If they ended with this first result as is commonly done in the case of the jockey, it is evident that the evacuations would enfeeble the most robust man, but the trainers soon pass to the second operation, the object of which is to develop the muscles, and to give more energy to the nutritive functions. These ends are obtained by gradual and regular exercise combined with a proper system of alimentation. He who is to run is not fed like the person prepared for boxing. The first is allowed only a small quantity of aliment, rather exciting than substantial. For the second, choice is made of such aliment as under small volume, furnishes to the organs, materials essentially nutritive, that is to say, after having evacuated all the subcutaneous useless parts, the nutritive action is directed to the muscles; these now almost solely engage attention.

Finally, the moral dispositions are objects of particular care. The person being trained is always accompanied by the trainer, whose object is to amuse the former with pleasant and agreeable stories and to keep away from him all circumstances which may excite anger or impatience. In a word he is taught *sang-froid*, courage, equanimity, qualities as necessary in the combat as muscular force itself.

After so many testimonies accumulated by M. Royer-Collard on the efficacy of training, nobody can now doubt the efficacy of this art, which consists in our seizing in some manner on the nutritive power, and directing it to a determinate object; to change sometimes in one sense, sometimes in another, the intimate structure of organs without employing any other means but that of the regimen. This principle once admitted and well understood, how many forms or different degrees of health could be happily modified by a systematic regimen which would only require patience and submission.

Marion county, June, 1844.

